# Supplementary material for: The Professional and Psycho-Emotional Impact of the COVID-19 Pandemic on Medical Care—A Romanian GPs’ Perspective
Source: Int J Environ Res Public Health. 2021 Feb 19;18(4):2031. doi: 10.3390/ijerph18042031 (PMC7922693; doi:10.3390/ijerph18042031)
Supplement: Supplementary file 1 [file ijerph-18-02031-s001.pdf]

Concentrarea/Distribuirea puterii sociale

Scoruri la nivel de județe

(0) Distribuirea puterii — Concentrarea puterii (100)

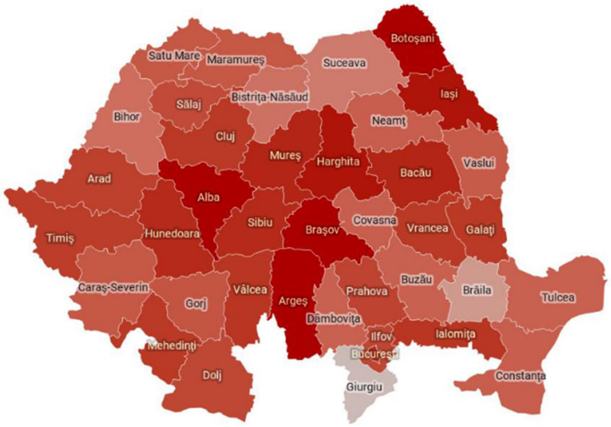

Notă. Reprezentare grafică a distribuției geografice a dimensiunii culturale Concentrarea/Distribuirea puterii sociale pe teritoriul României (N=3025). O nuanță mai apropiată de culoarea roșie indică un scor mai ridicat privind Concentrarea puterii sociale. Scorurile teoretice ale scalei variază între 0 și 100.

Hartă: D. David, M. Bartucz, S. Matu, M. Comșa (2020) • Creat cu Datawrapper

Colectivism/Autonomie (Individualism)

Scoruri la nivel de județe

(0) Colectivism — Autonomie (100)

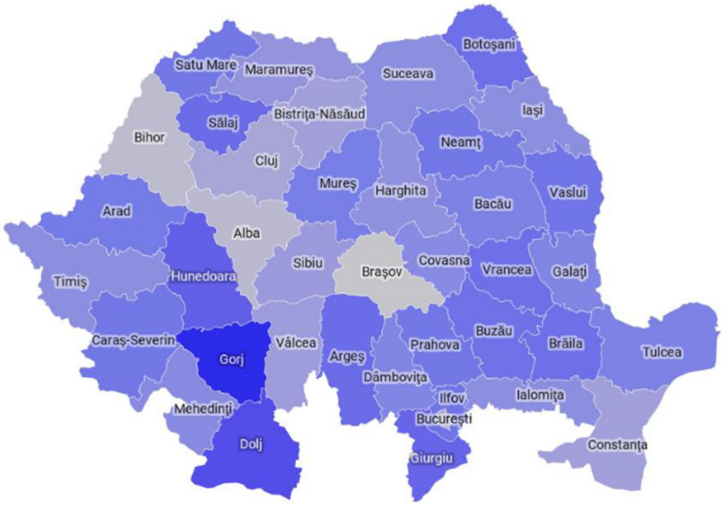

Notă. Reprezentare grafică a distribuției geografice a dimensiunii culturale Colectivism/Autonomie (Individualism) pe teritoriul României (N=3025). O nuanță mai apropiată de culoarea roșie indică un scor mai ridicat privind Autonomia (Individualismul). Scorurile teoretice ale scalei variază între 0 și 100.

Hartă: D. David, M. Bartucz, S. Matu, M. Comșa (2020) • Creat cu Datawrapper

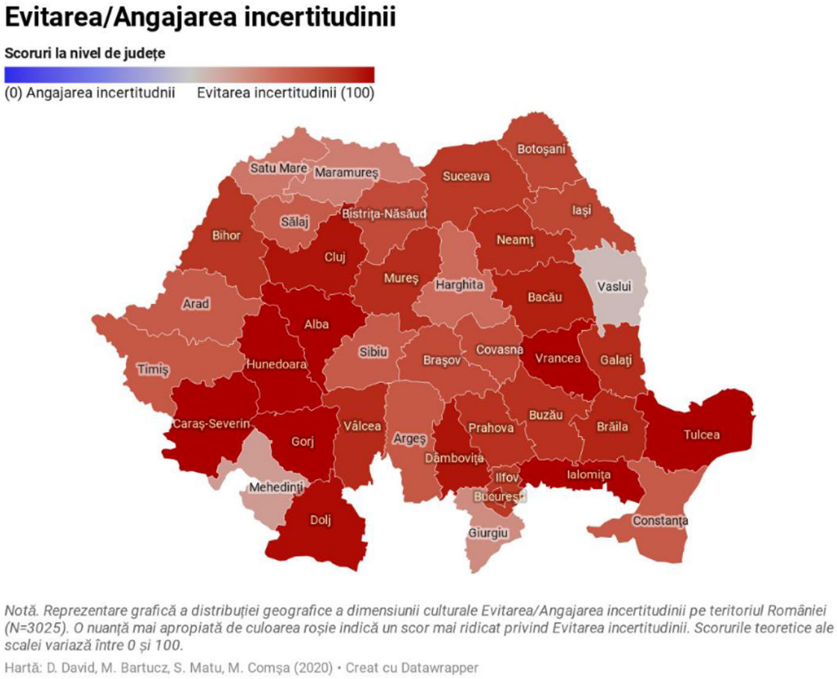

Figure S1. Distribution of participants by regions of Romania.

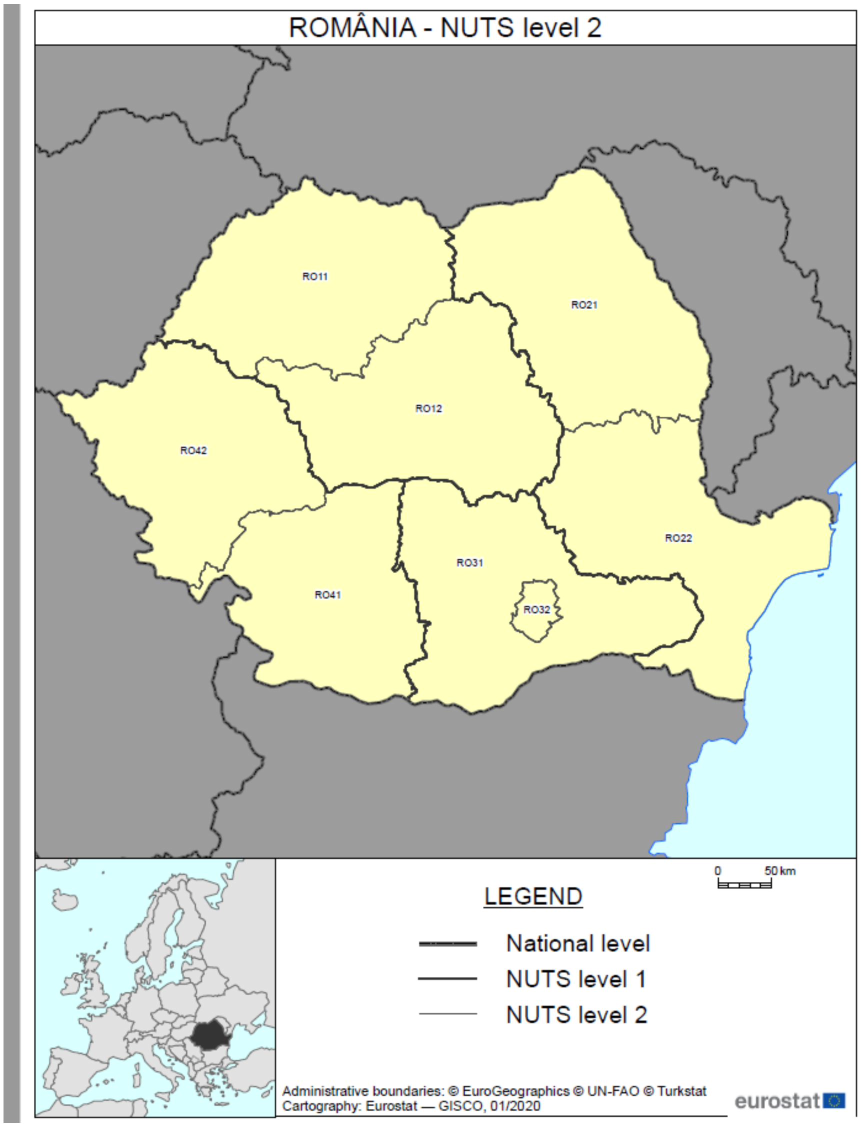

Figure S2. EUROSTAT Romania map NUTS II.
